# Effects of cytochrome P450 oxidoreductase genotypes on the pharmacokinetics of amlodipine in healthy Korean subjects

**DOI:** 10.1002/mgg3.1201

**Published:** 2020-03-05

**Authors:** Ji Min Han, Jeong Yee, Jee Eun Chung, Kyung Eun Lee, Kyungsoo Park, Hye Sun Gwak

**Affiliations:** ^1^ College of Pharmacy and Graduate School of Pharmaceutical Sciences Ewha Womans University Seoul Republic of Korea; ^2^ College of Pharmacy Hanyang University Ansan Republic of Korea; ^3^ College of Pharmacy Chungbuk National University Cheongju‐si Republic of Korea; ^4^ Department of Pharmacology Yonsei University College of Medicine Seoul Republic of Korea

**Keywords:** amlodipine, CYP3A, pharmacokinetics, *POR* polymorphism

## Abstract

**Background:**

The aim of this study was to investigate the effects of P450 oxidoreductase (*POR*) genetic polymorphisms on the pharmacokinetic parameters of amlodipine.

**Methods:**

After a single 10‐mg dose of amlodipine administration, 25 healthy male subjects completed genotyping for 12 single nucleotide polymorphisms (SNPs) of the *POR* genes, cytochrome P450 (CYP)3A4 g.25343G>A (CYP3A4*1G), and CYP3A5 g.12083G>A (CYP3A5*3). Stratified analysis and in silico analysis to predict the possible effects of given variants on splicing were performed.

**Results:**

The maximum blood concentration (C_max_) of amlodipine in carriers of g.57332T>C and g.56551G>A SNPs of the *POR* gene was statistically significantly different. In addition, T‐allele carriers of g.57332T>C had a 21% higher C_max_ than those with the CC genotype (*p* = .007). Subjects who carried the wild‐type g.56551G>A allele also had a 1.12‐fold significantly higher C_max_ than subjects with mutant‐type homozygous carriers (*p* = .033). In stratified analyses, g.57332T>C was significantly associated with a 1.3‐fold increase in C_max_ value in T‐allele carriers compared with subjects with the CC genotype in *CYP3A4* and *CYP3A5* expressers. POR g.57332T>C increased the score above the threshold in both ESEfinder 3.0 and HSF 3.1.

**Conclusion:**

This study identified a novel SNP of the *POR* gene, which affected amlodipine metabolism and may reduce interindividual variation in responses to amlodipine.

## Graphical abstract

Our study identified a novel SNP of the POR gene, which affected amlodipine metabolism and may reduce interindividual variation in response to amlodipine. Rs4732511 and rs7796654 SNPs of the POR gene showed statistically significant difference in the Cmax of amlodipine. After adjusting for CYP3A effects, rs4732511 was significantly associated with the difference in Cmax value.

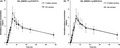

## 1 INTRODUCTION

Amlodipine is a 1,4‐dihydropyridine class of calcium channel blockers, and one of the most widely used agents for the treatment of angina and hypertension (Meredith & Elliott, 1992). It has high interindividual variation in blood pressure control, and polymorphisms of drug metabolism–related genes are one of the influencing factors (Fu et al., 2013; Kim et al., 2006). Clinical drug–drug interaction studies have shown that amlodipine acts as a substrate of the cytochrome P450 (CYP) 3A subfamily, especially CYP3A4 and CYP3A5, suggesting that its metabolism may be affected by changes in CYP3A metabolic activity (Glesby et al., 2005; Lee, Heeswijk, Alves, Smith, & Garg, 2011).

CYP3A is involved in 40%–50% of the oxidative biotransformation of current therapeutic agents (Evans & Relling, 1999). Because it is located in the intestinal mucosa and liver tissue, it is associated with drug metabolism ability after oral drug administration (Wilkinson, 1996). Of the four types of CYP3A, CYP3A4 and CYP3A5 are the most abundant in the liver and intestine in that order, whereas CYP3A7 and CYP3A43 are undetectable or expressed at very low levels in the adult liver (Burk et al., 2002; Gellner et al., 2001; Nelson et al., 1996; Westlind et al., 2001). CYP3A has large interindividual variability of 5‐ to 20‐fold in catalyzing drug metabolism, which are the substrates of CYP3A (Evans & Relling, 1999; Wilkinson, 1996). However, *CYP3A* polymorphism alone is not sufficient to account for the interindividual variation in CYP3A metabolic activity (Ingelman‐Sundberg, Sim, Gomez, & Rodriguez‐Antona, 2007; Özdemir et al., 2000).

The P450 oxidoreductase (*POR*, OMIM #124015) gene is a 78‐kDa microsomal protein containing both flavin adenine dinucleotide (FAD) and flavin mononucleotide (FMN) moieties, and is located on chromosome 7q11.23 containing 16 exons (Miller, Huang, Agrawal, & Giacomini, 2009). It affects CYP activity by donating electrons that are needed for CYP‐mediated substrate oxidation from nicotinamide adenine dinucleotide phosphate (NADPH) to microsomal (Type II) CYP450 enzymes (Masters, 2005). The importance of the *POR* gene in drug metabolism was reported in a study using liver‐specific knockout mice (Henderson et al., 2003). *POR* has highly polymorphic properties, suggesting that it might be responsible for the variation in metabolic activity among individuals (Agrawal, Choi, Giacomini, & Miller, 2010). Several studies have shown that *POR* polymorphisms affect the activity of CYP isoenzymes (Agrawal et al., 2010; De Jonge, Metalidis, Naesens, Lambrechts, & Kuypers, 2011; Elens et al., 2013; Huang, Agrawal, Giacomini, & Miller, 2008; Oneda et al., 2009). In addition, *POR* polymorphisms have been shown to more greatly influence the variation in CYP3A activity than CYP3A polymorphisms in Caucasian patients (Oneda et al., 2009).

Even though *POR* polymorphisms have important effects on interindividual variation in CYP activity, few studies have investigated these polymorphisms and their effects on amlodipine pharmacokinetics (PKs). Thus, the aim of this study was to investigate the effects of *PO*R gene polymorphisms on the PK parameters of amlodipine.

## 2 MATERIAL AND METHODS

### 2.1 Study populations

The study population was included from 50 healthy male volunteers who had participated in a bioequivalence study of a 10‐mg dose of amlodipine (Kim et al., 2013). Among these volunteers, 25 healthy men participated in this study after providing additional written consent for genotyping. Eligible subjects were men between the ages of 20 and 50 years, who were within 20% of their ideal body weight with no congenital abnormality or chronic disease. Exclusion criteria were as follows: (a) use of prescription drugs or herbal medications within 2 weeks or use of nonprescription drugs within 1 week before the study, which had the potential to interact with amlodipine; and (b) use of drugs that induce or inhibit drug‐metabolizing enzymes within 1 month before the study, which had the potential to interact with study medications. Vital signs monitoring, physical examination, and routine laboratory tests were performed before the start of the study.

### 2.2 Clinical study

The PK data of the study population were obtained from a previous single‐dose study of amlodipine (Kim et al., 2013). Subjects took a 10‐mg tablet of amlodipine orally with 240‐mL water at 8 a.m. after an overnight fast for 10 hr. Venous blood samples were collected into ethylenediaminetetraacetic acid‐containing tubes by an indwelling catheter inserted into the forearm at 0 (predose) and 0.5, 1, 1.5, 2, 3, 4, 6, 8, 10, 12, 16, 24, and 48 hr after dosing. Blood samples for genotyping were also collected, and genotyping was performed after the end of the study.

### 2.3 Editorial policies and ethical considerations

The study protocol was approved by the Ethics Committee of the Institutional Review Board (IRB No. 2012‐4‐0283). Informed consent was obtained from all patients before study participation.

### 2.4 Analysis of amlodipine concentrations and genotyping

Plasma amlodipine concentrations were analyzed using a validated ultra‐performance liquid chromatography tandem mass spectrometry method, as previously reported (Kim et al., 2013). Genomic DNA was prepared from blood samples using the QIAamp DNA Blood Mini Kit (QIAGEN GmbH,), according to the standard manufacturer's recommended procedures. To select *POR* (NM_000941.3) single nucleotide polymorphisms (SNPs), genetic information on the *POR* gene was incorporated into the Haploview Program (Jeong, Lee, Jeong, Chang, & Gwak, 2015). There were 74 SNPs in the *POR* gene, with a minor allele frequency (MAF)> 20% in Japanese and Han Chinese populations. Linkage disequilibrium blocks were constructed following the D’‐method in Haploview (Gabriel et al., 2002). Tagger function within Haploview was used to assign tag SNPs. A total of 12 SNPs were selected by adding 1 SNP (NG_008930.1:g.76686G>A) from a previously published study to 11 tag SNPs (NM_000941.3:c.1508C>T (p.(Ala503Val)), NG_008930.1:g.57332T>C, NG_008930.1:g.37537C>T, NG_008930.1:g.56228G>A, NG_008930.1:g.61444T>C, NM_000941.3:c.387A>G/T (p.(Pro129=)), NG_008930.1:g.53506T>C, NG_008930.1:g.40071T>C, NG_008930.1:g.56551G>A, NG_008930.1:g.5036A>C, and NG_008930.1:g.44272C>G) in the *POR* to capture common variations within the gene and the surrounding area with a minimum r^2^ of 0.80 (Ma et al., 2017). Additionally, NG_008421.1:g.25343G>A (CYP3A4*1G) and NG_007938.1:g.12083G>A (CYP3A5*3) were selected based on previous studies and Asian frequency (Danielak et al., 2017; Fukushima‐Uesaka et al., 2004; Kim, Park, Lee, Kang, & Park, 2007; Park et al., 2006; Park, Seo, Ahn, Kim, & Park, 2009; Yuan, Zhang, Deng, Wu, & Xiang, 2011; Zhou et al., 2011). Genotyping of *CYP3A4*,* CYP3A5,* and *POR* polymorphisms was conducted by a single‐base primer extension assay using ABI PRISM SNaPshot Multiplex Kits (ABI, Foster City, CA, USA) according to the manufacturer's recommendations.

### 2.5 PK analysis

PK parameters were calculated using actual sampling times. Maximum blood concentration (C_max_) and time to maximum concentration (T_max_) were determined by searching the observed data. The area under the plasma concentration–time curve from time zero to the time of the last concentration (AUC_last_) was calculated using the linear trapezoidal rule. The AUC from time zero to infinity (AUC_inf_) was the sum of AUC_last_ and C_last_/k_e_, where C_last_ is the last quantifiable concentration and k_e_ is the terminal elimination rate constant; the half‐life was 0.693/k_e_. Plasma concentrations during the terminal phase were fitted to a log linear line by the least squares method to obtain the k_e_. PK parameters were analyzed by a noncompartmental method using WinNonlin5.3 (Pharsight Corporation).

### 2.6 In silico analyses

To predict the possible effects of given variants on splicing, different computational tools were used. Netgene2 and Splice Site Prediction by Neural Network (NNSPLICE) were used for splice site predictions (Brunak, Engelbrecht, & Knudsen, 1991; Reese, Eeckman, Kulp, & Haussler, 1997). Alternations of the splicing factor‐binding site pattern caused by the given mutation were evaluated by using Exonic Splicing Enhancer (ESE) finder 3.0 and Human Splicing Finder (HSF) 3.1 (Cartegni, 2003; Desmet et al., 2009). We used the default threshold values, and a score for a given sequence was considered to be potentially significant if it was above the threshold values.

### 2.7 Statistical analysis

All PK data were expressed as the mean ± standard deviation (*SD*). Hardy–Weinberg equilibrium (HWE) was tested using the chi‐square test. Differences in PK parameters among the genotype groups were evaluated using the Mann–Whitney rank sum test for two‐group comparisons. Stratified analyses were conducted to investigate the effects of *POR* gene polymorphisms on amlodipine PKs using CYP3A expressers. *p* values < .05 were considered statistically significant. Statistical analyses were performed using SPSS 20.0 (International Business Machines Corp.).

## 3 RESULTS

The mean age, weight, and height of the subjects were 26.8 ± 5.9 years, 67.9 ± 8.2 kg, and 174.2 ± 4.8 cm, respectively. The mean PK parameter values were as follows: C_max_: 6.09 ± 1.06 ng/ml, T_max_: 6.32 ± 0.75 hr, half‐life: 40.75 ± 7.29 hr, AUC_last_: 257.45 ± 54.99 hr·μg/mL, AUC_inf_: 267.80 ± 59.71 hr·μg/mL, ke: 0.02 ± 0.00 hr^‐1^, oral clearance (CL/F): 39.25 ± 9.35 L/h, and volume of distribution (Vd/F): 2,278.51 ± 560.01 L. All SNPs were in accordance with HWE. The MAFs of g.57332T>C and g.56551G>A were 0.125 and 0.24, respectively.

**Figure 1:**
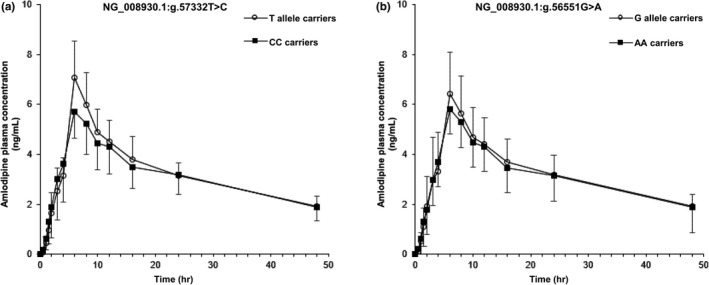
Mean (±*SD*) amlodipine plasma concentrations after oral administration of single 10‐mg dose of amlodipine in healthy subject according to *POR* genotype. (a) Mean amlodipine plasma concentration according to genotype of g.57332T>C. (b) Mean amlodipine plasma concentration according to genotype of g.56551G>A

Table 1 described the association between polymorphisms of *POR* genes and PK parameter values. The locations of the 12 selected SNPs in the *POR* gene were the intron region (*n* = 8), 5’‐ untranslated region (UTR; *n* = 1), 3’‐UTR (*n* = 1), missense region (*n* = 1), and synonymous region (*n* = 1). Of the 12 *POR* SNPs, g.57332T>C and g.56551G>A were significantly associated with the C_max_ of amlodipine. T‐allele carriers of g.57332T>C had a 21% higher C_max_ than those with the CC genotype (*p* = .007). Subjects who carried the wild‐type allele of g.56551G>A also showed a 1.12‐fold significantly higher C_max_ than subjects with mutant‐type homozygous carriers (*p* = .033). The mean (±*SD*) plasma concentration–time profiles of amlodipine after oral administration according to genotypes of g.57332T>C and g.56551G>A are shown in Figure 1. There was no statistically significant difference between *CYP3A* polymorphisms and PK parameters of amlodipine.

**Table 1:** Differences in amlodipine pharmacokinetic parameters among *CYP3A4*,* CYP3A*5*,* and *POR* genotypes in study population

Gene polymorphism		Grouped genotype (Patient %)	C_max_ (ng/ml)	T_max_ (hr)	Half‐life (hr)	k_e_ (hr^−1^)	AUC_last_ (hr·μg/ml)	AUC_inf_ (hr·μg/ml)	CL/*F* (L/hr)	Vd/*F* (L)	Location
CYP3A4	g.25343G>A	GG (68.0)	6.18 ± 1.25	6.24 ± 0.66	40.42 ± 8.12	0.02 ± 0.00	254.43 ± 61.64	264.61 ± 67.48	40.22 ± 10.61	2,303.09 ± 608.87	intronic
	(CYP3A4*1G)	GA,AA (32.0)	5.89 ± 0.49	6.50 ± 0.93	41.45 ± 5.55	0.02 ± 0.00	263.88 ± 40.17	274.57 ± 41.70	37.20 ± 5.97	2,226.28 ± 472.53	
		*p*‐value	.440	.628	.440	.440	.628	.511	.511	.711	
CYP3A5	g.12083G>A	AA (60.0)	6.27 ± 1.31	6.13 ± 0.52	40.45 ± 8.44	0.02 ± 0.00	257.29 ± 65.32	267.79 ± 71.50	40.05 ± 11.33	2,288.84 ± 638.02	splice donor
	(CYP3A5*3)	AG,GG (40.0)	5.81 ± 0.47	6.60 ± 0.97	41.21 ± 5.52	0.02 ± 0.00	257.70 ± 37.77	267.81 ± 39.44	38.07 ± 5.58	2,263.02 ± 450.13	
		*p*‐value	.144	.338	.531	.531	.978	.892	.892	.978	
POR	c.1508C>T, p.(Ala503Val)	CC,CT (60.0)	5.96 ± 1.19	6.27 ± 0.70	41.76 ± 7.56	0.02 ± 0.00	258.53 ± 58.03	269.51 ± 62.90	39.33 ± 10.70	2,345.16 ± 651.78	missense
	(POR*28)	TT (40.0)	6.28 ± 0.86	6.40 ± 0.84	39.23 ± 6.98	0.02 ± 0.00	255.84 ± 53.12	265.23 ± 57.79	39.14 ± 7.42	2,178.53 ± 396.19	
		*p*‐value	.605	.807	.238	.238	.765	.807	.807	.765	
	g.57332T>C	TT,CT (25.0)	6.95 ± 0.88	6.00 ± 0.00	44.89 ± 11.89	0.02 ± 0.00	285.99 ± 56.03	302.28 ± 65.99	34.50 ± 7.93	2,149.91 ± 320.31	intronic
		CC (75.0)	5.76 ± 0.98	6.44 ± 0.86	39.61 ± 5.00	0.02 ± 0.00	248.41 ± 54.47	257.01 ± 56.57	40.84 ± 9.71	2,333.25 ± 633.14	
		*p*‐value	.007**	.454	.137	.137	.156	.119	.119	.537	
	g.37537C>T	CC,CT (92.0)	6.23 ± 0.92	6.26 ± 0.69	40.73 ± 7.61	0.02 ± 0.00	259.22 ± 49.35	269.66 ± 54.42	38.47 ± 7.31	2,225.91 ± 417.75	intronic
		TT (8.0)	4.51 ± 1.69	7.00 ± 1.41	41.04 ± 1.20	0.02 ± 0.00	237.18 ± 134.50	246.42 ± 139.31	48.30 ± 27.31	2,883.40 ± 1,700.54	
		*p*‐value	.167	.427	.480	.480	.733	.733	.733	.807	
	g.56228G>A	GG, GA (48.0)	6.05 ± 0.98	6.17 ± 0.58	42.42 ± 8.26	0.02 ± 0.00	268.52 ± 53.49	280.71 ± 59.11	37.18 ± 8.19	2,243.47 ± 481.33	intronic
		AA (52.0)	6.12 ± 1.18	6.46 ± 0.88	39.22 ± 6.20	0.02 ± 0.00	247.24 ± 56.48	255.88 ± 60.06	41.17 ± 10.25	2,310.86 ± 642.25	
		*p*‐value	.769	.538	.225	.225	.470	.470	.470	1.000	
	g.61444T>C	TT, CT (72.0)	6.14 ± 1.22	6.33 ± 0.77	41.15 ± 7.34	0.02 ± 0.00	265.58 ± 61.71	276.63 ± 66.72	38.47 ± 10.65	2,255.46 ± 632.44	intronic
		CC (28.0)	5.95 ± 0.49	6.29 ± 0.76	39.73 ± 7.62	0.02 ± 0.00	236.57 ± 24.49	245.08 ± 28.49	41.27 ± 4.69	2,337.78 ± 339.79	
		*p*‐value	.657	.929	.270	.270	.297	.326	.326	.389	
	c.387A>G/T, p.(Pro129=)	AA (44.0)	6.06 ± 1.18	6.55 ± 0.93	40.76 ± 5.80	0.02 ± 0.00	257.23 ± 65.31	267.21 ± 69.01	40.00 ± 11.58	2,335.41 ± 710.35	synonymous
		AT, AG, TT,GG (56.0)	6.11 ± 1.01	6.14 ± 0.53	40.74 ± 8.50	0.02 ± 0.00	257.63 ± 47.97	268.26 ± 54.01	38.67 ± 7.59	2,233.80 ± 431.19	
		*p*‐value	.687	.403	.687	.687	.979	.936	.936	.936	
	g.53506T>C	TT,CT (84.0)	6.16 ± 1.14	6.38 ± 0.80	41.16 ± 7.90	0.02 ± 0.00	260.85 ± 57.39	271.68 ± 62.38	38.86 ± 9.84	2,272.90 ± 590.72	intronic
		CC (16.0)	5.70 ± 0.41	6.00 ± 0.000	38.62 ± 1.30	0.02 ± 0.00	239.59 ± 41.58	247.40 ± 43.77	41.33 ± 6.89	2,307.95 ± 425.65	
		*p*‐value	.231	.592	.642	.642	.452	.409	.409	.642	
	g.40071T>C	TT,CT (68.0)	6.24 ± 1.02	6.35 ± 0.79	41.42 ± 8.53	0.02 ± 0.00	262.70 ± 54.08	273.96 ± 59.77	38.11 ± 7.99	2,236.76 ± 466.63	intronic
		CC (32.0)	5.78 ± 1.14	6.25 ± 0.71	39.32 ± 3.55	0.02 ± 0.00	246.31 ± 58.95	254.70 ± 61.39	41.69 ± 12.00	2,367.23 ± 751.20	
		*p*‐value	.475	.842	.798	.798	.711	.588	.588	.932	
	g.56551G>A	GG,GA (44.0)	6.47 ± 1.32	6.36 ± 0.81	41.67 ± 9.37	0.02 ± 0.00	268.40 ± 62.98	280.43 ± 69.94	38.17 ± 11.70	2,240.43 ± 672.00	intronic
		AA (56.0)	5.79 ± 0.73	6.29 ± 0.73	40.03 ± 5.41	0.02 ± 0.00	248.85 ± 48.48	257.87 ± 50.77	40.11 ± 7.38	2,308.43 ± 478.96	
		*p*‐value	.033*	.893	.647	.647	.244	.244	.244	.317	
	g.5036A>C	AA,AC (92.0)	6.15 ± 0.90	6.26 ± 0.69	40.74 ± 7.61	0.02 ± 0.00	259.01 ± 49.03	269.54 ± 54.25	38.48 ± 7.30	2,227.02 ± 416.21	5'‐UTR
		CC (8.0)	5.34 ± 2.86	7.00 ± 1.41	40.89 ± 1.41	0.02 ± 0.00	239.50 ± 137.78	247.77 ± 141.22	48.19 ± 27.47	2,870.69 ± 1718.51	
		*p*‐value	.733	.427	.600	.600	.880	.807	.807	.880	
	g.44272C>G	CC,CG (92.0)	6.23 ± 0.92	6.26 ± 0.69	40.73 ± 7.61	0.02 ± 0.00	259.22 ± 49.35	269.66 ± 54.42	38.47 ± 7.31	2,225.91 ± 417.75	intronic
		GG (8.0)	4.51 ± 1.69	7.00 ± 1.41	41.04 ± 1.20	0.02 ± 0.00	237.18 ± 134.50	246.42 ± 139.31	48.30 ± 27.31	2,883.40 ± 1,700.54	
		*p*‐value	.167	.427	.480	.480	.733	.733	.733	.807	
	g.76686G>A	GG,GA (72.0)	6.03 ± 1.15	6.22 ± 0.65	41.22 ± 7.70	0.02 ± 0.00	256.07 ± 54.28	266.66 ± 59.22	39.50 ± 9.98	2,318.14 ± 598.44	3'‐UTR
		AA (28.0)	6.24 ± 0.85	6.57 ± 0.98	39.55 ± 6.49	0.02 ± 0.00	261.01 ± 61.04	270.73 ± 65.66	38.62 ± 8.17	2,176.60 ± 472.14	
		*p*‐value	.701	.534	.495	.495	.929	1.000	1.000	.745	

Abbreviations: AUC_inf_, area under the plasma concentration–time curve from time zero to the infinity; AUC_last_, area under the plasma concentration–time curve from time zero to the time of the last concentration; CL, clearance; C_max_, maximum plasma concentration; F, bioavailability; ke, elimination rate constant; T_max_, time to maximum concentration; Vd, volume of distribution.

*p < .05, **p < .01.

Stratified analyses were performed for the association between PK parameter values and *POR* polymorphisms in *CYP3A* expressers (Table 2). *POR* SNPs that were statistically significant in univariate analysis were included. POR g.57332T>C was significantly associated with the 1.3‐fold increase in C_max_ value in T‐allele carriers compared with subjects with the CC genotype in *CYP3A4* and *CYP3A5* expressers.

**Table 2:** Amlodipine pharmacokinetic parameters according to the *POR* genotypes in subjects with wild‐type homozygotes of *CYP3A4* and *CYP3A5*

Gene polymorphism		Grouped genotype (Patient %)	C_max_ (ng/ml)	T_max_ (hr)	Half‐life (hr)	k_e_ (hr^−1^)	AUC_last_ (hr·μg/ml)	AUC_inf_ (hr·μg/ml)	CL/*F* (L/hr)	Vd/*F* (L)
Wild‐type homozygotes of g.25343G>A (CYP3A4*1G)										
*POR*	g.57332T>C	TT,CT (18.8)	7.73 ± 0.15**	6.00 ± 0.00	46.92 ± 18.22	0.02 ± 0.01	301.35 ± 81.31	321.81 ± 95.21	33.43 ± 11.92	2,105.21 ± 467.12
		CC (81.2)	5.78 ± 1.13**	6.31 ± 0.75	39.22 ± 4.38	0.02 ± 0.00	244.02 ± 57.40	252.15 ± 59.72	41.87 ± 10.57	2,367.08 ± 662.55
*POR*	g.56551G>A	GG,GA (41.2)	6.57 ± 1.68	6.57 ± 0.98	41.99 ± 11.70	0.02 ± 0.00	266.82 ± 80.17	279.57 ± 89.01	39.62 ± 14.74	2,315.78 ± 841.94
		AA (58.8)	5.91 ± 0.84	6.00 ± 0.00	39.32 ± 4.75	0.02 ± 0.00	245.75 ± 47.62	254.14 ± 50.16	40.64 ± 7.39	2,294.21 ± 431.60
Wild‐type homozygotes of g.12083G>A (CYP3A5*3)										
*POR*	g.57332T>C	TT,CT (21.4)	7.73 ± 0.15*	6.00 ± 0.00	46.92 ± 18.22	0.02 ± 0.01	301.35 ± 81.31	321.81 ± 95.21	33.43 ± 11.92	2,105.21 ± 467.12
		CC (78.6)	5.83 ± 1.23*	6.18 ± 0.60	39.04 ± 4.13	0.02 ± 0.00	246.03 ± 62.63	254.21 ± 65.18	41.93 ± 11.58	2,359.28 ± 711.11
*POR*	g.56551G>A	GG,GA (40.0)	6.78 ± 1.74	6.33 ± 0.82	43.17 ± 12.35	0.02 ± 0.00	272.00 ± 86.53	285.99 ± 95.71	39.31 ± 16.12	2,353.65 ± 915.74
		AA (60.0)	5.94 ± 0.88	6.00 ± 0.00	38.63 ± 4.48	0.02 ± 0.00	247.48 ± 50.17	255.65 ± 52.97	40.54 ± 7.83	2,245.64 ± 427.81

Abbreviations: AUC_inf_, area under the plasma concentration–time curve from time zero to the infinity; AUC_last_, area under the plasma concentration–time curve from time zero to the time of the last concentration; CL, clearance; C_max_, maximum plasma concentration; F, bioavailability; ke, elimination rate constant; T_max_, time to maximum concentration; Vd, volume of distribution.

a *p < .05, **p < .01.

Analysis of two SNPs (g.57332T>C and g.56551G>A) with Netgene2 and NNSPLICE showed a significant association with the C_max_ of amlodipine, and did not show the presence of an altered splicing donor or acceptor. However, the results generated by ESEfinder 3.0 indicated that the g.57332T>C increased the score of a SRSF1 (IgM‐BRCA1) from −0.873 to 2.232 (threshold: 1.867), creating a new high score‐enhancing motif. HSF 3.1, with a different scoring algorithm from that of ESEfinder 3.0, also showed that this mutation led to the formation of two SF2/ASF (IgM‐BRCA1)‐enhancing motifs (CTCCCCG and CCCCGCT); the scores were 75.31 and 73.31, respectively (threshold: 70.51) (Supplement Table S1).

## 4 DISCUSSION

The main result of this study was that g.57332T>C and g.56551G>A of the *POR* genes showed statistically significant differences in the C_max_ of amlodipine. After adjusting for CYP3A effects, g.57332T>C remained a significant factor for amlodipine PKs.

*POR* is a gene that gives electrons to CYP450 enzymes (Masters, 2005). The structure of the *POR* gene consists of an NADPH‐docking site and FAD in one lobe and FMN and P450‐interacting domain in the other lobe (Wang et al., 1997). The electron transfer proceeds from NADPH to FAD, followed by FMN, and finally to P450. In a study that examined the effects of 35 *POR* variants on CYP enzymes, it was revealed that the *POR* variants are involved in the activity of the CYP enzymes, although the effects vary depending on the variants (Agrawal, Huang, & Miller, 2008).

The association between *POR* polymorphisms and CYP3A4 metabolic activities using different substrates including midazolam, testosterone, erythromycin, and quinidine was assessed (Agrawal et al., 2010). This study revealed that the impact of *POR* polymorphisms on CYP3A4 activity was substrate specific, possibly due to the substrate‐induced conformational changes in CYP3A4. In the case of the most common variant, c.1508C<T (POR*28), the polymorphism reduced the CYP3A4 activity to 61%–77% of wild‐type with testosterone and midazolam while it had similar activity to wild‐type with quinidine and erythromycin. Our study failed to show significant differences in any PK parameters of amlodipine according to genotypes of POR*28. Instead, novel *POR* variants, g.57332T>C and g.56551G>A, which affected C_max_ value of amlodipine, were identified. In stratified analyses to rule out the effects of *CYP3A* polymorphisms and find *POR* variant effects, g.57332T>C remained significant SNP in both *CYP3A4* and *CYP3A5* expressers. In particular, the difference in C_max_ values according to the g.57332T>C genotypes in expressers increased (20.7% vs. around 33%).

The SNPs of g.57332T>C and g.56551G>A are located in intronic regions, which are thought to not be involved in protein production. However, intronic regions have the potential to affect mRNA splicing and alter protein expression or activity; thus, analyzing these regions may be useful (Pagani & Baralle, 2004; Raponi & Baralle, 2010). To evaluate the splicing effects of these two SNPs, we employed various computational tools and found that g.57332T>C affected the splicing of ‐POR. In addition, it has been revealed that intronic POR variants also affected CYP activity. An in vitro study evaluating the effect of POR polymorphisms on CYP activity showed that three intronic SNPs were associated with alteration of various CYP functions (Gomes et al., 2009). The intronic SNP g.18557G>A decreased CYP3A4 activity, whereas g.25676C>T and g.30986G>A increased microsomal activities of CYP1A2, 2C8, 2C19, and 3A4 and of CYP2C19 and 3A4, respectively. Therefore, g.57332T>C may be a candidate SNP that affects the amlodipine absorption rate.

CYP3A4 and CYP3A5 are major enzymes of CYP3A family (Zhu et al., 2014). CYP3A5 g.12083G>A (CYP3A5*3), which was analyzed in this study, is the most common CYP3A5 polymorphic form and causes a truncated CYP3A5 enzyme and loss of CYP3A5 activity (Kim et al., 2007; Kuehl et al., 2001; Park et al., 2006; Park et al., 2009). However, in an amlodipine PK study, subjects with CYP3A5*3/*3 had a lower plasma concentration of amlodipine than subjects who carried CYP3A5*1 (Kim et al., 2006). In terms of CYP3A4, g.25343G>A (CYP3A4*1G) has a high frequency in Asian populations (Fukushima‐Uesaka et al., 2004; Zhou et al., 2011). In a study that demonstrated the impact of g.25343G>A (CYP3A4*1G) polymorphism on fentanyl metabolism, plasma concentration in the *1G/*1G group was significantly higher than that in the *1/*1G and *1/*1 groups, indicating loss‐of‐function mutation (Zhou et al., 2011). In contrast, a clinical study using clopidogrel showed similar PK profiles between wild‐type homozygotes and mutant allele carriers (Danielak et al., 2017). As described above, the effects of CYP3A4 and CYP3A5 polymorphisms on drug PKs are not consistent. In this study, no significant effects of CYP3A4 and CYP3A5 polymorphisms including g.12083G>A (CYP3A5*3) and g.25343G>A (CYP3A4*1G) on the PK parameters of amlodipine were observed.

Only the C_max_ among PK parameters was statistically significant; however, in applying these results to clinical settings, we need to recognize that failure to achieve statistical significance does not necessarily mean clinical insignificance. With respect to g.57332T>C in *CYP3A4* and *CYP3A5* expressers, the difference in AUC_inf_ and CL/F was more than 25% according to genotypes, but statistical significance was not found. This was possibly due to the small sample size, resulting in an underpowered study.

There were some limitations in this study. The sample size was too small to obtain statistically significant results. Our study population only comprised males, so it was impossible to analyze gender differences. Multiple testing correction was not performed to avoid the possible loss of true positives.

In conclusion, to the best of our knowledge, this is the first study to evaluate the association between *POR* gene polymorphisms and amlodipine PKs in a Korean population. The identified novel SNP of the *POR* gene, which was shown to affect amlodipine metabolism, may be useful for reducing interindividual variation in responses to amlodipine. However, these results should be interpreted with caution due to the risk of false‐positive results. Additional studies are needed to verify the results of this study.

## CONFLICT OF INTEREST

The authors declare to have no conflict of interest.

## AUTHORS' CONTRIBUTIONS

JMH, KP, and HSG conceived and design of study. JMH, JEJ, KEL, and HSG made acquisition and analysis of data. JMH and JY made an interpretation of data. JMH, KP, and HSG have been involved in drafting the manuscript. KP and HSG have been involved in revising the manuscript. All authors approved the manuscript to be published.

## Supporting information

Supplementary Material
