# Supplementary material for: Effects of cytochrome P450 oxidoreductase genotypes on the pharmacokinetics of amlodipine in healthy Korean subjects
Source: Mol Genet Genomic Med. 2020 Mar 5;8(5):e1201. doi: 10.1002/mgg3.1201 (PMC7216797; doi:10.1002/mgg3.1201)
Supplement: Supplementary file 1 — Supplementary Material [file MGG3-8-e1201-s001.docx]

**Supplement 1.** Potential splicing regulatory sequences in wild-type and mutant-type of g.56551G>A and g.57332T>C

by ESEfinder 3.0 and Human Splicing Finder (HSF) 3.1.

|  | Linked SR or motif (threshold) | Wild-type sequence (Score) | Mutant-type Sequence (Score) | Variation |
| --- | --- | --- | --- | --- |
| ESE finder 3.0 | | | | |
| g.56551G>A | SRp40 (2.67) | CCGCTGC (2.271) | CCACTGC (4.879^*^) |  |
| g.57332T>C | SRp55 (2.676) | TGCTTC (3.832^*^) | TGCTCC (1.227) |  |
|  | SRSF1 (IgM-BRCA1) (1.867) | CTTCCCG (0.412) | CTCCCCG (2.481^*^) |  |
|  | SRSF1 (IgM-BRCA1) (1.867) | TCCCGCT (-0.873) | CCCCGCT (2.232^*^) |  |
| Human Splicing Finder 3.1 | | | | |
| g.56551G>A | SC35^a^ (75.05) | GTCCGCTG (90.53) | GTCCACTG (93.12) | +2.85% |
|  | SRp40^a^ (78.08) |  | CCACTGC (91.26) | New site |
|  | SF2/ASF (IgM-BRCA1)^a^ (70.51) | CGCTGCA (72.92) | CACTGCA (70.69) | -3.06% |
|  | PESE Octamers^b^ |  | ACTGCAGA (29.29) | New site |
|  | 9G8^c^ (59.245) |  | GTCCAC (60.47) | New site |
|  | FAS-ESS hexamers^d^ | AGTCCG |  | Broken |
| g.57332T>C | SRp55^a^ (73.86) | TGCTTC (81.35) |  | Broken |
|  | SC35^a^ (75.05) | GCTTCCCG (78.67) | GCTCCCCG (80.09) | +1.8% |
|  | SF2/ASF (IgM-BRCA1)^a^ (70.51) |  | CTCCCCG (75.31) | New site |
|  | SF2/ASF (IgM-BRCA1)^a^ (70.51) |  | CCCCGCT (73.31) | New site |
|  | Motif 3^e^ (60) | GCTTCCCG (65.86) | GCTCCCCG (83.04) | +26.09% |
|  | Motif 3^e^ (60) | CTTCCCGC (69.98) |  | Broken |
|  | IIEs^f^ | ATGCTT |  | Broken |

Note: ^*^Scores above the threshold

Predicted based on ^a^ESE Finder matrices

^b^Predicted Exonic Splicing Enhancers

^c^Human Splice Finder matrices based on experimental data

^d^Fluorescence-Activated Screen for Exonic Splicing Silencers

^e^Silencer motifs from Sironi et al

^f^Intron Identify Elements
